# Risk assessment of space radiation-induced invasive cancer in mouse models of lung and colorectal cancer

**DOI:** 10.1093/jrr/rrt149

**Published:** 2014-03

**Authors:** Sang Bum Kim, Aadil Kaisani, Jerry W. Shay

**Affiliations:** Department of Cell Biology, UT Southwestern Medical Center, 5323 Harry Hines Blvd, Dallas, TX 75390, USA

**Keywords:** simulated solar particle events, LA-1 K-Ras, CPC;Apc, chronic inflammation

## Abstract

Lung cancer accounts for the most cancer-related deaths worldwide, estimated 1.3 million deaths each year. We have chosen to use a lung cancer susceptible (LA-1 K-Ras) mouse model [ 1] to examine the effects of heavy ion (^56^Fe−) and simulated solar particle events (SPEs) on cancer progression. The long-term goal of the study is to assess the risk of developing invasive cancers in this mouse model and to extrapolate the data to human risk projections [ 2]. The murine model (LA-1) randomly expresses mutated *K-RAS* in a subset of lung cells resulting in initiation and formation of lesions that mimic lung cancer progression in humans. Greater than 50% of the mice with oncogenic K-ras expression die in less than a year with a small percentage of mutant mice living to a maximum of ∼600 days. About 9% of LA-1 mice spontaneously develop invasive non-small cell lung adenocarcinomas. We have compared this lung cancer susceptible mouse model with another mouse model of susceptibility to colorectal cancer (CRC) [ 3]. About 6% of CDX2P-Cre, APC^+/loxP^ (CPC;Apc) mice spontaneously develop invasive cancers. Although the risk of normal mice to tumorigenesis upon exposure to low and high linear energy transfer radiation has been studied in the past, very limited data are available on progression of cancer-susceptible mice to more advanced, perhaps fatal, invasive cancers.

Initial studies included administrating whole-body proton irradiation as a simulated SPE of 2.0 Gy over 2 h with a wide range of energies (50–150 MeV). Histopathological analysis of the irradiated LA-1 and CPC;Apc mice 1 year post-ionizing radiation (IR) demonstrated a progression in tumor grade (in the lung from 9 to 19% and in the colon from 6 to 21%). Thus, 2.0 Gy SPE demonstrated a significant increase in the progression of invasive adenocarcinomas in the lung and colon. In previous studies using the LA-1 mouse model 70 days post-fractionated 1.0 Gy 1 GeV/n ^56^Fe− IR, there was an increased expression of inflammatory factors within the lung (∼200 days prior to the observation of invasive cancer). In studies on the CPC;Apc mice 100 days post-SPE irradiation, there was an increased expression of senescence-associated inflammatory genes in tumor-free distal colon tissues. These data suggested that chronic inflammation may be important in the progression of invasive cancer. These results indicate that exposure to simulated SPE can increase the risk of invasive adenocarcinoma progression in lung and colorectal cancer.
